# Comprehensive comparison of polysaccharides from *Ganoderma lucidum* and *G. sinense*: chemical, antitumor, immunomodulating and gut-microbiota modulatory properties

**DOI:** 10.1038/s41598-018-22885-7

**Published:** 2018-04-18

**Authors:** Li-Feng Li, Hong-Bing Liu, Quan-Wei Zhang, Zhi-Peng Li, Tin-Long Wong, Hau-Yee Fung, Ji-Xia Zhang, Su-Ping Bai, Ai-Ping Lu, Quan-Bin Han

**Affiliations:** 1 School of Chinese Medicine, Hong Kong Baptist University, Hong Kong, China; 2 School of Pharmacy, Xinxiang Medical University, Xinxiang, China

## Abstract

Both *Ganoderma lucidum* (GL) and *G. sinense* (GS) are used as Lingzhi in China. Their functions are assumed to mainly derive from triterpenes and polysaccharides; however, the two species have very different triterpenes profiles, if this was the case, then the bioactivity of these two species should differ. Instead, could the polysaccharides be similar, contributing to the shared therapeutic basis? In this study, two main polysaccharide fractions from different batches of GL and GS were systematically compared by a series of chemical and biological experiments. The results showed that the polysaccharides from two species shared the same structural features in terms of mono-/oligo-saccharide profiles, molecular size, sugar linkages, and IR/NMR spectra. In addition, these polysaccharides showed similar tumor-suppressive activity in mice. Further study on RAW264.7 cells indicated that these polysaccharides exhibited similar inducing effects to macrophages, as evaluated in the phagocytosis function, NO/cytokines production, inhibition against the viability and migration of cancer cells. Mechanistic investigation revealed the identical activation via TLR-4 related MAPK/NF-κB signaling pathway and gut-microbiota modulatory effects. In summary, GL and GS polysaccharides presented similar chemical features, antitumor/immunomodulating activities and mechanism; this establishes polysaccharides as the active principles and supports the official use of both species as Lingzhi.

## Introduction

Lingzhi is a functional food and herbal medicine well-known for its anti-carcinogenic properties^1,2^. In the Chinese Pharmacopeia, Lingzhi is described as the fruiting body of two species: *Ganoderma lucidum* (GL) and *G. sinense* (GS). To date, the investigations of Lingzhi’s antitumor activities focus on two groups of chemicals: triterpenes and polysaccharides. Triterpenes called ganoderic acids have been found cytotoxic towards a variety of cancer cell lines^3,4^, and therefore are assumed to be responsible for the antitumor activity. However, these cytotoxic triterpenes show abundance only in GL^5,6^. If ganoderic acids are responsible for Lingzhi’s biological activity, then GS—having few and in small amounts—would not have been used through the centuries as functionally equivalent to GL. Beside ganoderic acids, GL polysaccharides are also associated with the antitumor effect because they showed immune-modulatory activities of inhibiting DNA polymerase, inhibiting post-translational modification of the Ras oncoprotein^7,8^, or inducing antibodies to tumor-associated Globo H-series epitopes^9^. if polysaccharides are responsible for Lingzhi’s biological activity, then GS and GL, having the similar range and amounts of polysaccharides, could be used as more or less equivalent. This possibility is further supported by the fact that Lingzhi is typically used in water decoctions. Polysaccharides are hydrophilic, hence abundant in decoctions, while triterpenes are not. Therefore, we hypothesized the polysaccharides contributed to the shared use of the Lingzhi decoctions traditionally used in both homes and clinics.

**Figure 1:**
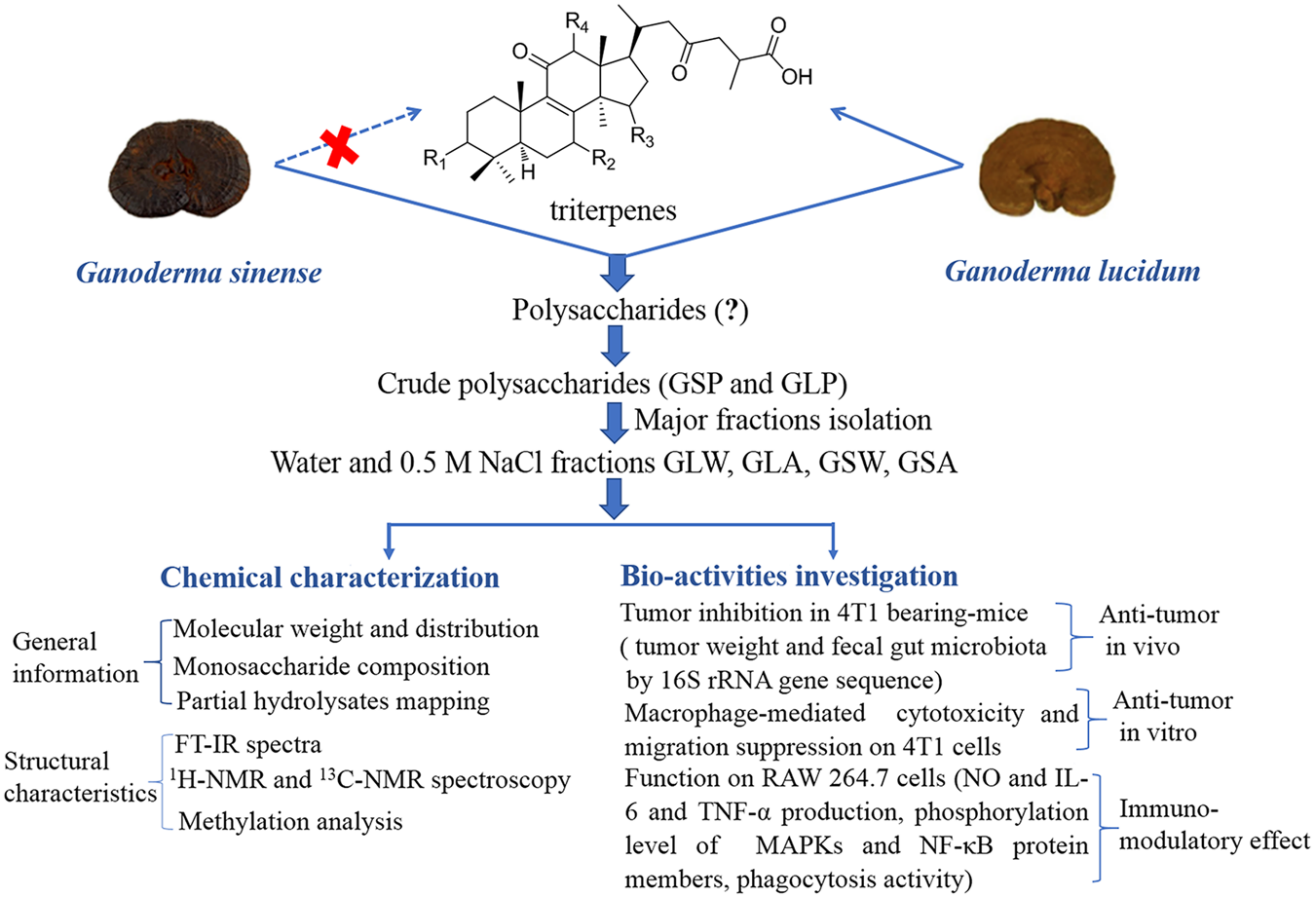
Research scheme and methodology.

Recent studies have compared the crude polysaccharides or aqueous extracts from the two *Ganoderma* species. It was found that polysaccharides from GS and GL were similar not only in molecular weight distribution but also in basic effects on lymphocytes and macrophages *in vitro*^10–12^. However, systematic information on structural similarity is limited as literatures regarding the chemistry of GS polysaccharides are far fewer than GL polysaccharides. In addition, to our knowledge, whether GS polysaccharides have comparable antitumor effect with GL polysaccharides has not been proved *in vivo*, not to say the possible mechanism of action. Therefore, systematic investigation on polysaccharides was thus imperative in this study to decipher their similarity in chemical and bioactive properties. As shown in Fig. 1, using eight batches of two major polysaccharide fractions-GLW and GLA isolated from crude polysaccharides of GL, and seven batches of GSW and GSA from GS, we determined their chemical similarity. We then evaluated these polysaccharides’ antitumor effects *in vivo* and *in vitro*. Finally, we took insight into exploring the potential mechanism of action by analyzing fecal gut microbiota and macrophage stimulation. The results of our studies provide comprehensive and strong evidence to support the use of GL and GS as Lingzhi in China.

## Results

### Comparative basic characterization of polysaccharides

According to our previous study, *Ganoderma* polysaccharides mainly have two major fractions (accounting for >85%). They are water fraction and sodium (0.5 mol/L) fraction obtained from ion-exchange chromatography. As the further purified polysaccharides from water/sodium fraction showed closely similar chemistry, chemical comparison in this study will focus on these two major fractions, in terms of the molecular weight distribution pattern, monosaccharide profiles produced by complete hydrolysis, and oligosaccharides profiles produced by partial hydrolysis. We prepared multiple batches of these two fractions from eight *G. lucidum* samples and seven *G. sinense* samples. The water fractions, were labeled GLW (n = 8) and GSW (n = 7), and the sodium fractions, were named GLA (n = 8) and GSA (n = 7).

**Figure 2:**
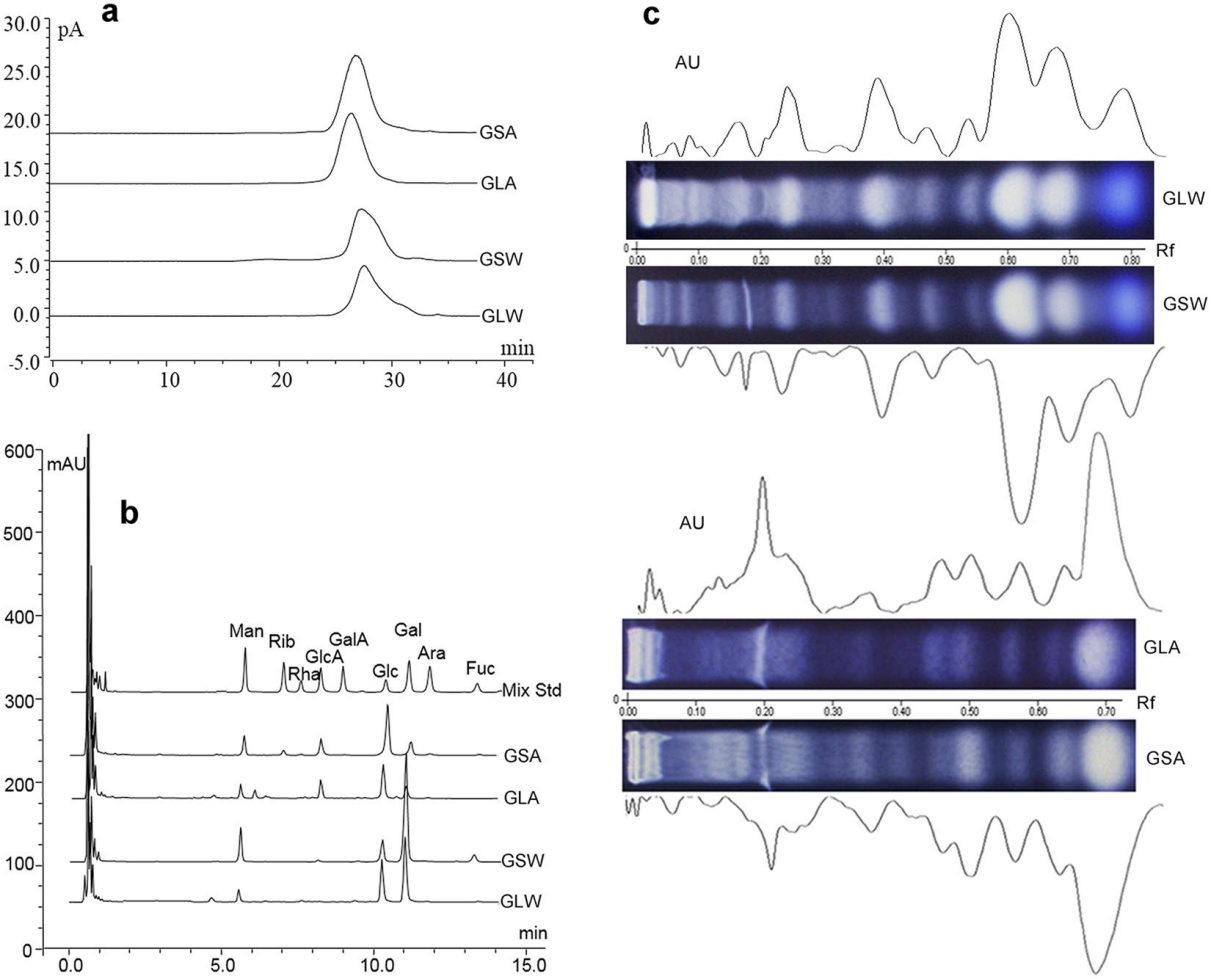
General chemical characterization of GLW, GSW, GLA and GSA. (**a**) Typical HPGPC chromatograms of molecular weight distribution; (**b**) Typical monosaccharide compositional profiles. Monosaccharides were detected by UPLC-UV (λ = 245 nm) after 1-phenyl-3-methyl-5-pyrazolone (PMP) derivatization Mixed standards (Mix Std) contained the following saccharides: Mannose (Man); Ribose (Rib); Rhamnose (Rha); Glucuronic acid (GlcA); Galacturonic acid (GalA); Glucose (Glc); Galactose (Gal); Arabinose (Ara); Fucose (Fuc); (**c**) Typical TFA induced partial hydrolysates profiles containing oligosaccharides by HPTLC analysis; Samples were applied on 0.2 mm silica gel 60 HPTLC plates (Merck, Germany) with an automatic TLC sampler (CAMAG, Switzerland). Then the plate was developed with n-butanol–ethanol–water 5: 3: 2: (v/v) and colorized with 5% H_2_SO_4_ in ethanol solution and heated to make bands colored clearly. Then the plate was photographed and scanned by a TLC visualizer and scanner (CAMAG, Switzerland).

The molecular weight distribution was determined by high-performance gel permeation chromatography (HPGPC). The results were summarized in Fig. 2a and Supp. Figure 1. As calculated by using the established molecular weight-retention time calibration curve, the molecular sizes of GLW and GSW fell in a narrow range of 13.4–17.0 kDa. GLA and GSA presented larger molecular sizes of 17.6–23.3 kDa and 15.1–22.6 kDa, respectively.

The monosaccharides profiles got by complete acid hydrolysis of GLW are summarized in Supp. Figure 2, suggesting eight batches of samples from different sources showed generally similar patterns. This is also true to GSW, GLA and GSA (Supp. Figure 2). To check the similarity between GLW and GSW, GLA and GSA, one typical chromatogram in each fractional polysaccharide is shown in Fig. 2b. The results demonstrate that sugar composition pattern exhibited high consistency between GLW and GSW, regardless of the enhancing fucose (Fuc) proportion in the latter. GLA and GSA had similar sugar compositional profiles except GSA has more ribose (Rib).

The partial acid hydrolysates containing oligosaccharides of the polysaccharides were investigated using high performance thin layer chromatography (HPTLC). In method validation process, GLW-2, GSW-2, GLA-2 and GSA-2 as well as dextran (Mw = 20 kDa close to these tested polysaccharides) as a polysaccharide standard were used to repeat hydrolyzation and developing solvent optimization. Standards of maltopentaose (Rf = 0.19), sucrose (Rf = 0.65) and glucose (Rf = 0.70) were used to show oligosaccharide spots distribution region. Overall, the mapping showed similar spotting patterns among eight batches of GLW (Supp. Figure 3). The very similar result can be found in GSW, GLA and GSA (Supp. Figure 3). The typical images and scanning chromatograms are presented in Fig. 2c. The GLW and GSW showed similar patterns of partial hydrolysate although there were some differences in content; GLA and GSA were quite similar with each other.

### Comparative structural information of polysaccharides

The obtained result suggested that polysaccharides from two *Ganoderma* species presented basic similarity, then comparison went to structure relation properties in terms of methylation analysis, and IR/NMR spectral analysis.

**Figure 3:**
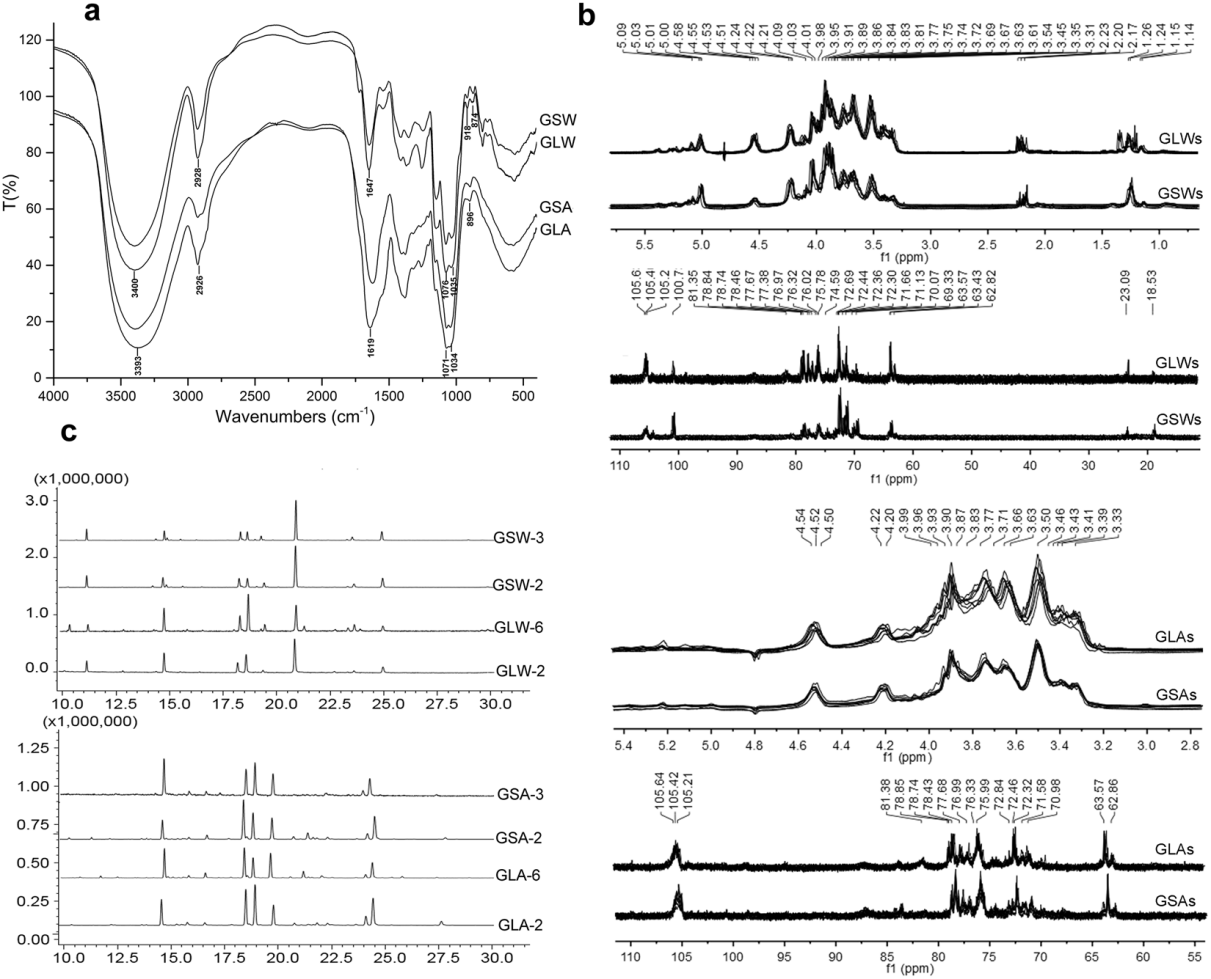
Structural related characterization of GLW, GSW, GLA and GSA. IR spectra (**a**) were got by KBr pellet method with the scan range from 4000 to 400 cm^−1^ and typical spectra were shown. ^1^H-NMR and ^13^C-NMR (**b**) spectra were recorded at 400 MHz and 100 MHz, respectively; Linkage types distribution chromatograms (**c**) were got by GC-MS analysis after methylation.

FT-IR spectroscopy demonstrated that very similar IR spectral patterns had been achieved between eight batches of GLWs (Supp. Figure 4). The case is also true to GSWs, GLAs and GSAs (Supp. Figure 4). Typical spectra (Fig. 3a) are shown for comparison between GLW and GSW, GLA and GSA. All fractions presented common absorptions of functional group of polysaccharides, such as a band of about 3400 cm^−1^ corresponding to O-H stretching vibrations, 2900 cm^−1^ corresponding to C-H stretching, and 1050 cm^−1^ corresponding to -C-O- group. Both GLA and GSA spectra had a band near 890 cm^−1^, which can be probably attributed to absorption for β-glycosidic linkage. Bands near 918 cm^−1^ and 874 cm^−1^ probably corresponding to α-glycosidic linkage can be observed in both GLW and GSW^12^.

The ^1^H-NMR and ^13^C-NMR spectra (Fig. 3b) showed that GLWs, GSWs, GLAs and GSAs reached individual consistency among multiple sample batches. Then comparison went to GLW and GSW, GLA and GSA. We found that GLW and GSW had similar resonance patterns, despite some minor variety. Based on the data in the literature, major characteristics of GLW and GSW are as follows: the signal around *δ*_H_ 4.51~5.12 ppm and *δ*_C_ 100.7~105.6 ppm and was assigned to anomeric protons/carbons. Among them, α- and β-configuration both presented^13^. The weaker resonance at *δ*_C_ 23.1 and *δ*_H_ 2.20 was possibly due to the presence of N-Acetyl-D-glucosamine^14,15^. The signal at around *δ*_C_ 18.5 and *δ*_H_ 1.26 was due to the existence of Fuc^16,17^. As for GLA and GSA, they look identical. The signal crowded in a narrow region ranging from *δ*_H_ 4.51~4.56 ppm and *δ*_C_ 105.1~105.6 ppm was assigned to anomeric protons/carbons, suggesting the presence of β-anomeric configurations.

Methylation analysis was used to compare the major linkage types. As demonstrated in Fig. 3c, and supplementary Table 1, five main types of sugar linkage were revealed for GLW and GSW^16,18,19^: (1 → 3)-linked Glc*p*, (1 → 4)-linked Glc*p*, (1 → 6)-linked Gal*p*, (1 → 2,6)-linked Gal*p* and terminal Glc*p*. Significant similarity was also found in the results of GLA and GSA, which presented unique residues (1 → 3, 6)-linked Glc*p* and (1 → 6)-linked Glc*p*. All the results were confirmed by analyzing two batches of samples.

In summary, these results show that the polysaccharides from these two species have no significant differences in chemistry. The next step was to compare the similarity in antitumor effects and its possible mechanism related to macrophage and gut microbiota modulation. Since polysaccharides from different batches were highly consistent in GLWs, GSWs, GLAs and GSAs, one typical batch in each group was selected to perform bioassay.

### Polysaccharides from two species similarly suppressed the growth of 4T1 breast cancer in BALB/C mice

**Figure 4:**
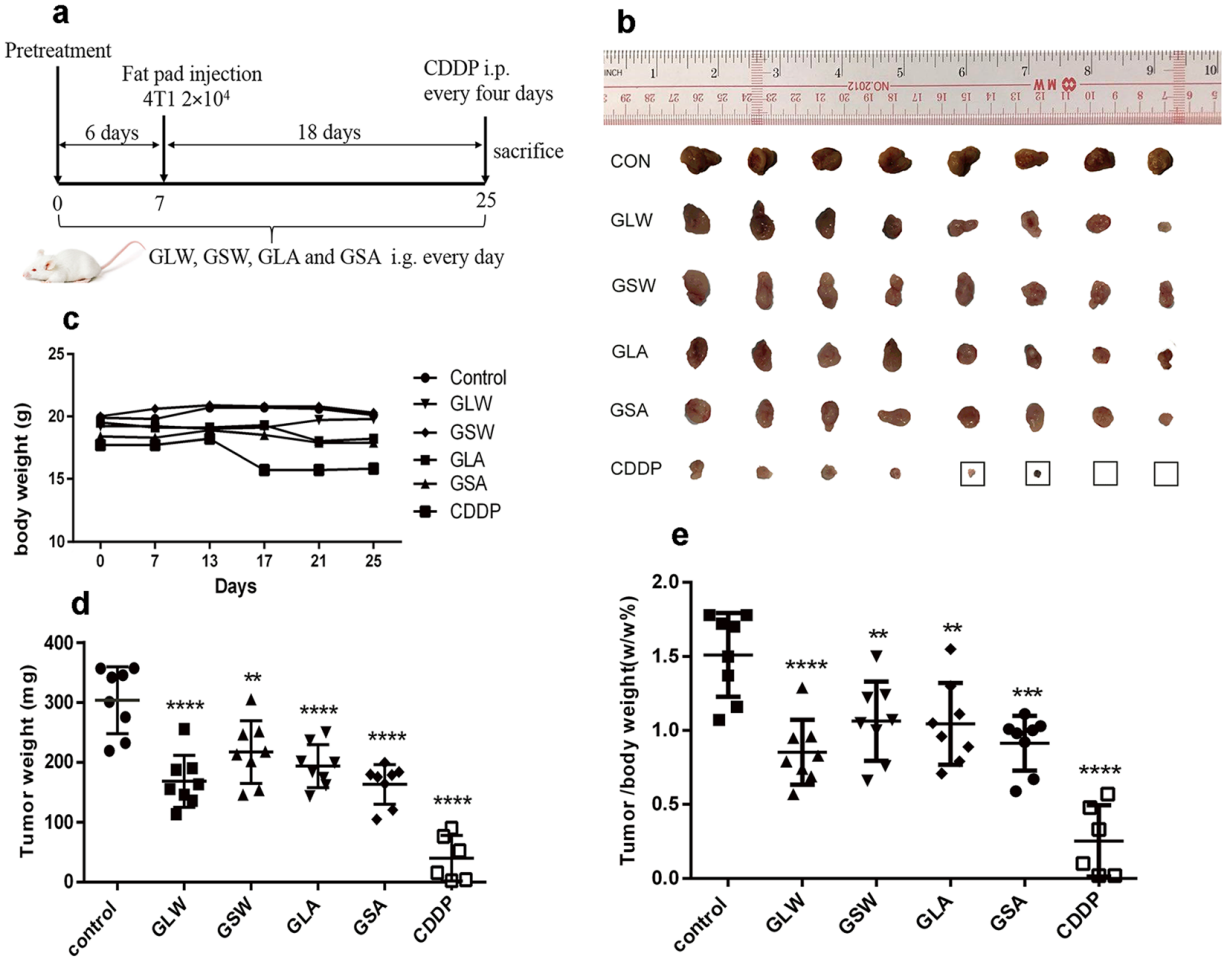
Polysaccharides inhibits carcinogenesis in 4T1-bearing BABL/C mice. 4T1 cells (2 × 10^4^) were implanted into mice mammary fat pad tissue (n = 8 in each group). After 6 days intragastrical administration in advance with the polysaccharides 200 mg/kg, mice were treated for another 18 days. Cis-Di-chiorodiamineplatinum (II) (CDDP) as positive control was intraperitoneally injected with 4.0 mg/kg CDDP every four days after 4T1 cells implantation. Control: 4T1 tumor bearing mice with water gavage; GLW, GSW, GLA and GSA: polysaccharide of GLW-2, GSW-2, GLA-2 or GSA-2 gavage treatment group, respectively. (**a**) Experiment process; (**b**) body weight; (**c**) tumor images captured by the end of experiment. The black frame label was denoted as the dead subject before the end of experiment; (**d**) tumor weight; (**e**) tumor weight/body weight. Data are shown as mean ± SD. Significant difference *p < 0.05, **p < 0.01, ***p < 0.001 compared with control group or GSA vs GLA, GSW vs GLW.

Using 4T1 breast cancer-bearing BALB/C mice (experiment course is shown in Fig. 4a), our study demonstrated that all polysaccharides from the two species exerted significant (*p* < 0.01) and similar suppressing effects on tumor growth. Briefly, the tumor weights in the GLW, GSW, GLA and GSA-treated 4T1-bearing mice were significantly reduced to 168.7 ± 43.4 mg, 217.3 ± 52.3 mg, 193.8 ± 36.2 mg and 163.4 ± 33.0 mg, respectively compared to the mice in control group (303.8 ± 56.0 mg) (Fig. 4b,d). In addition, there was still no significant difference between GLW and GSW group, GLA and GSA group when the result was recalculated and described by the ratio of tumor weight accounting for body weight (Fig. 4e). Meanwhile, comparing to traditional chemotherapy drug Cis-Di-chiorodiamineplatinum (II) (CDDP), a positive control in our study, all polysaccharides have less toxicity with tumor suppression as CDDP induced dramatic drop of body weight (Fig. 4c) and mass mortality.

### Polysaccharides from two species induced macrophage’s effect on cell viability and migration inhibition on 4T1 cells *in vitro*

**Figure 5:**
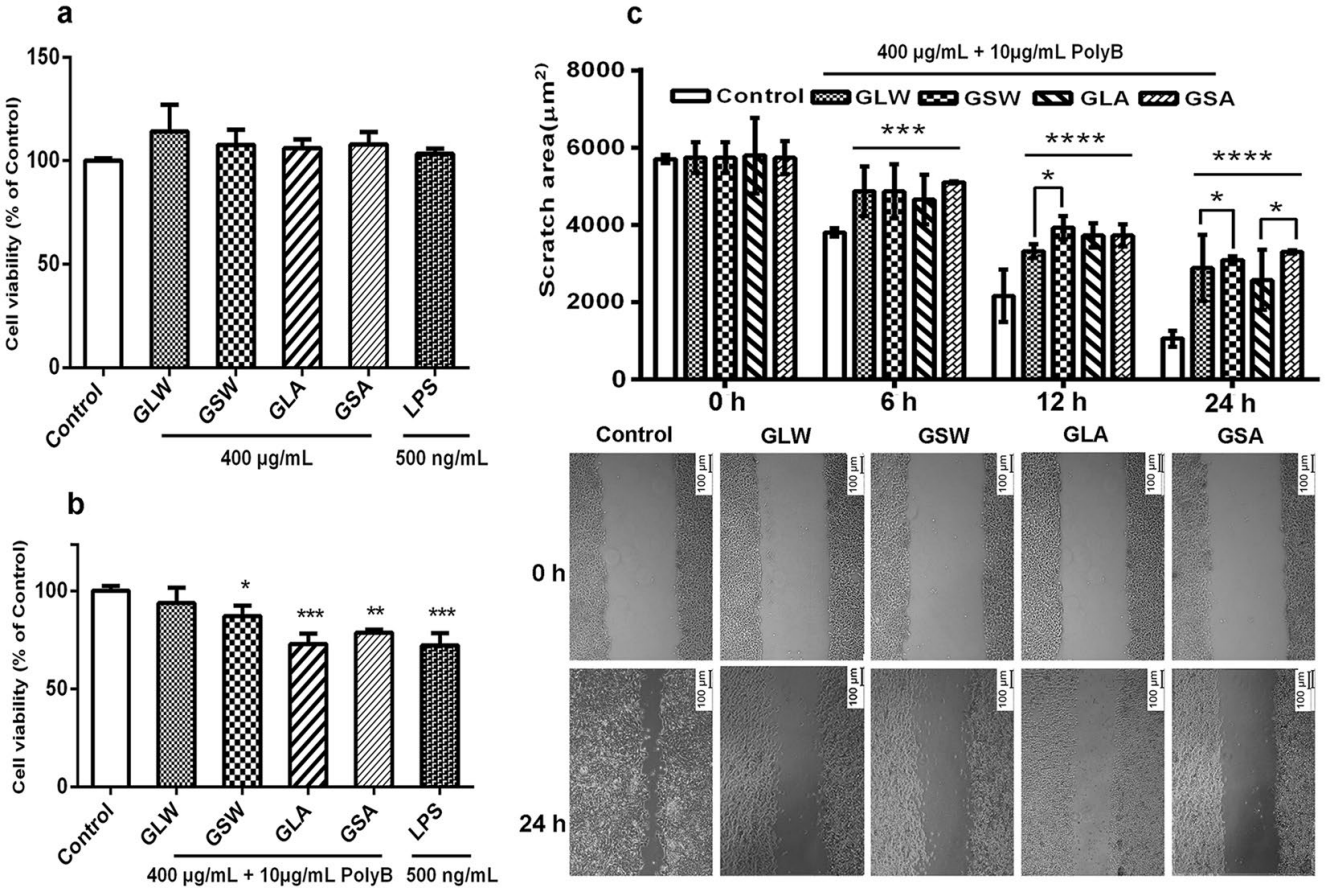
Effect of the polysaccharides from two species on cell viability by MTT assay and suppressing migration by scratch wound healing assay in 4T1 cell. (**a**) 4T1 cells were treated with 400 μg/mL GLW-2, GSW-2, GLA-2 and GSA-2 or 500 ng/mL LPS for 24 h; (**b**) 4T1 cells were exposed to supernatants of RAW264.7 cells after treatment with above sample with 10 μg/ml polymyxin B (PolyB) or 500 ng/mL LPS treatment for 24 h; (**c**) scratched 4T1 cells were treated with above supernatants of RAW264.7 cells incubated in DEME medium with 2% FBS. Images were photographed, and scratch distance was measured at 0, 6, 12, 24 h. All results are presented as mean ± SD. *P < 0.05; **P < 0.01; ***P < 0.001, ****P < 0.0001 compared with the control group or GSA vs GLA, GSW vs GLW.

As shown in Fig. 5a, GLW, GSW, GLA, GSA and LPS (positive control in RAW264.7 cell stimulation experiment) alone cannot directly affect the cell viability of 4T1 cells, compared to the control group (100%). However, the cell viability of 4T1 cells decreased when treated with the supernatants of these polysaccharides-treated RAW264.7 cells (Fig. 5b). Comparing GLW and GSW, GLA and GSA, the inhibition rates showed no significant difference.

As it is known that 4T1 cells exhibit higher metastasis, we performed scratch assays to further examine the effect of supernatants of RAW264.7 cells after polysaccharides treatment on the migration properties of 4T1 cells. The results (Fig. 5c) showed that, 6 h after treatment, all polysaccharides significantly (*P* < 0.001) reduced mobility of 4T1 cells *in vitro*, suggesting that they could similarly inhibit cell metastasis.

### Immunomodulatory stimulation effect on murine macrophage RAW264.7 cells

**Figure 6:**
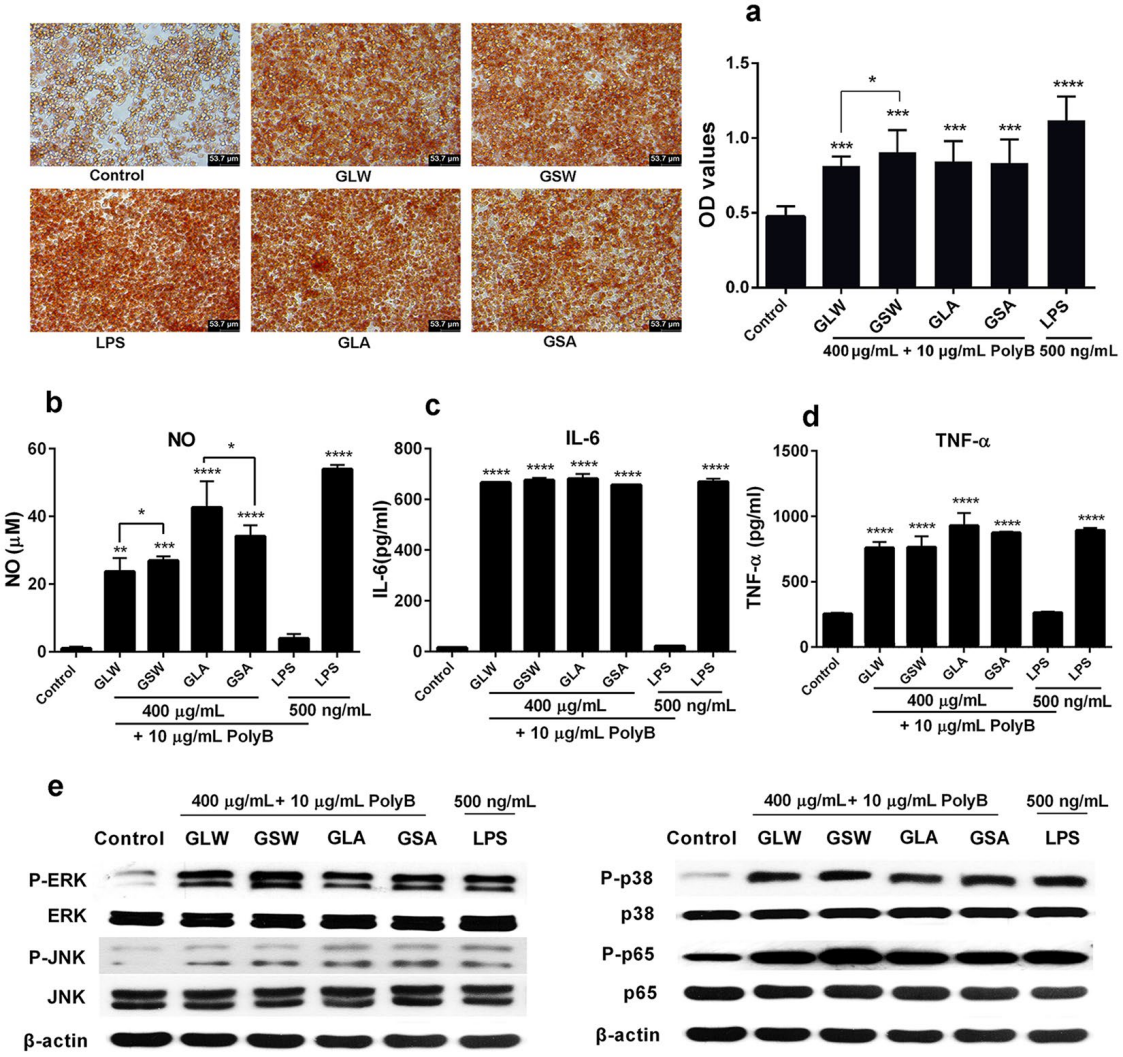
Effect of the polysaccharides from two species on activation on murine macrophage RAW264.7 cells. (**a**) Phagocytic activity. Seeded RAW264.7 cells were exposed to 400 μg/mL GLA-2, GSA-2, GLW-2 and GSW-2 with 10 μg/mL polymyxin B (PolyB) for 24 h, respectively. 0.1% neutral red was added as phagocytic substance. The phagocytosis of RAW 264.7 cells was observed under a microscope and the neutral red uptake measurement was at 570 nm; The production of NO (**b**), IL-6 (**c**) and TNF-α (**d**). Seeded RAW264.7 cells were treated with 400 μg/mL GLA-2, GSA-2, GLW-2 and GSW-2 with 10 μg/mL polymyxin B (PolyB), respectively and positive control LPS (500 ng/mL) with the presence or absence of PolyB for 24 h; (**e**) phosphorylation of ERK, JNK, and p38 MAPK and p65 in RAW264.7 macrophages cells after incubation with above sample for 30 min. All Western blots presented in were cropped to improve clarity. Full-length blots are displayed in Supplementary Figure 5; Data are presented as mean ± SD. *P < 0.05; **P < 0.01; ***P < 0.001, ****P < 0.0001 compared with the control group or GSA vs GLA, GSW vs GLW.

The result of phagocytosis test (Fig. 6a) showed that all polysaccharides from two species significantly promoted neutral red uptake on RAW264.7 cell, comparing with control group (*P* < 0.001). The GLW and GSW presented a significant difference (*P* < 0.05), while GLA and GSA were similar with each other.

**Figure 7:**
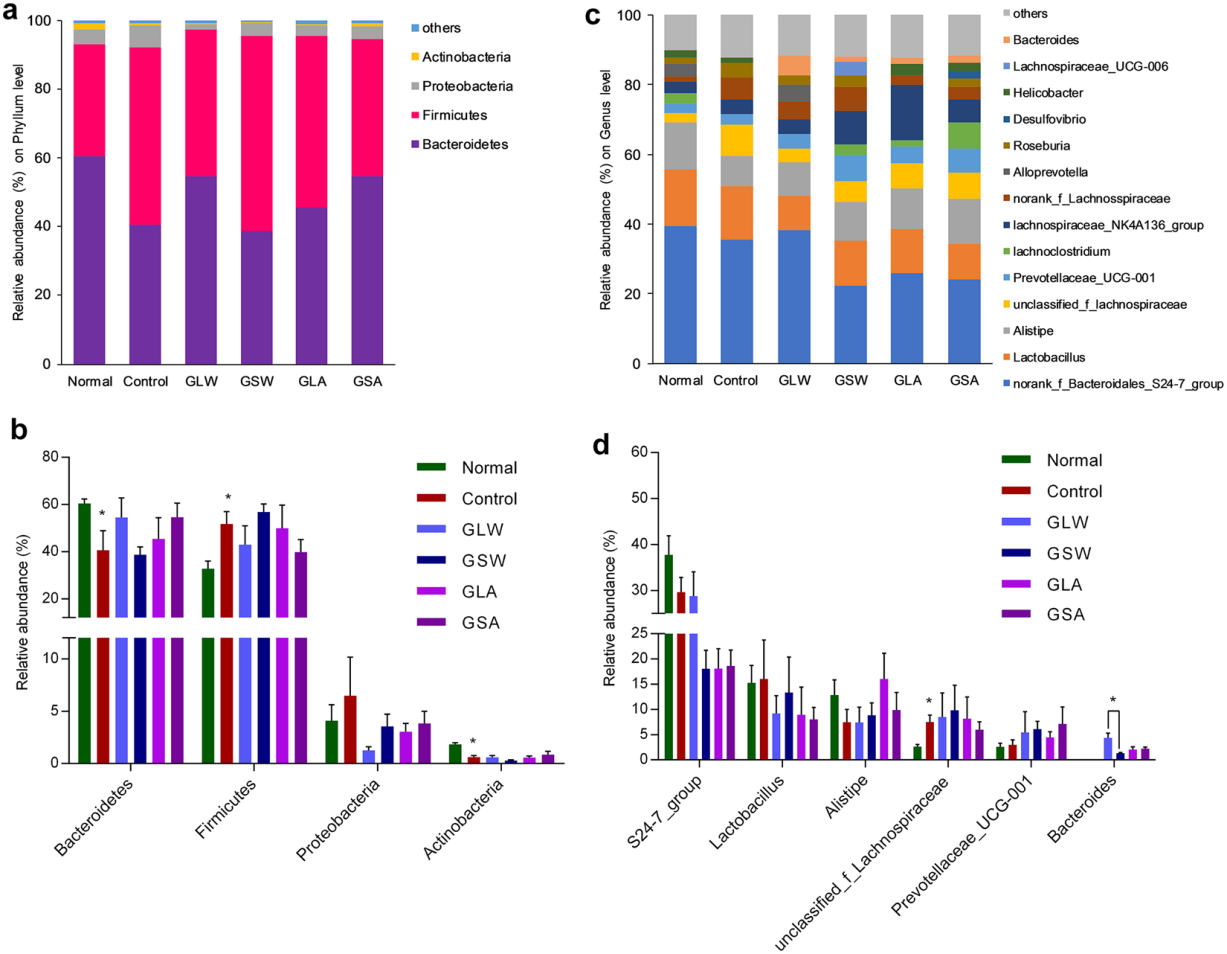
Taxonomic classification of gut microbiota in the six group mice by the relative abundances of phylum (**a**) and (**b**), genus (**c**) and (**d**) (n = 5); Normal: mice without tumor or any polysaccharide treatment; Control: 4T1 tumor bearing mice; GLW, GSW, GLA and GSA: polysaccharide of GLW-2, GSW-2, GLA-2 or GSA-2 treatment group, respectively. Data shown are the mean ± SD. Significant difference *p < 0.05, **p < 0.01, ***p < 0.001 compared with control group or GSA vs GLA, GSW vs GLW.

As shown in Fig. 6b, the production of NO increased significantly after treatment with GLA and GSA, reaching 42.6 ± 7.7 μΜ and 34.1 ± 3.2 μΜ (*P* < 0.0001), comparing with control. This was higher than GLW and GSW, for which NO production decreased to 23.7 ± 4.0 μΜ (*P* < 0.001) and 26.9 ± 1.3 μΜ (*P* < 0.01), respectively. A significant difference (*P* < 0.05) occurred between GLA and GSA, with greater NO production in the former. GLW and GSW had no significant difference in this regard. On the production of IL-6 (Fig. 7c), all polysaccharides exhibited similar effects, ranging from 657 to 682 pg/mL. The level of TNF-α (Fig. 7d) in cells treated with GLW and GSW showed a decreasing trend compared to GLA and GSA. No significant difference was found either between GLW and GSW or GLA and GSA.

Western blot assays were used for analyzing the activation of important MAPK and NF-κB protein members in RAW264.7 cells after incubation with typical fractional polysaccharides for 30 min. Our result showed all these polysaccharides clearly increase the phosphorylation level of phosphorylated ERK, JNK, p38 MAPK as well as of p65 (Fig. 6e).

### Effect on gut microbiota composition in 4T1 breast cancer-bearing mice

Taxonomy-based analysis indicated (Fig. 7a,b) a total of 4 phyla, being identified as *Firmicutes*, *Bacteroidetes*, *Proteobacteria* and *Actinobacteria*, accounted for about 99% of the total reads in all groups. Compared with the normal group, mice in control group contained significantly more *Firmicutes* (47.0%) and *Proteobacteria* (3.0%) but significantly less *Bacteroidetes* (48.5%) and *Actinobacteria* (0.38%) (*p* < 0.05). The ratio of *Firmicutes* to *Bacteroidetes* showed some reversed trends after treatment with these polysaccharides. At the genus level, we found *S24-7*, *Lactobacillus*, *Alistipes*, *unclassfied_f_Lahnospiraceae* and *Prevotellaceae_UCG-001* accounted for more than 60% of the total population in each group (Fig. 5c,d). Compared with the normal group, the abundance of *S24-7* and *Alistipes* showed a decreasing trend. A rising tendency was found in *unclassfied*_*f_Lahnospiraceae*. After polysaccharides treatment, the shift of *Alistipes* tended to be balanced. Two genera of *Prevotellaceae_UCG-001* and *Bacteroides* presented rising tendency; the latter was seldom found in normal and control group. Generally, our results indicated that GLW and GSW affected gut microbiota in tumor-bearing mice in a similar behavior. The similar case can also be found in mice fed with GLA and GSA.

## Discussion

Two species, *Ganoderma lucidum* and *G. sinense*, officially share the name of Lingzhi in China, and are used interchangeably. Chemically, these two species are both rich in polysaccharides but differ greatly in triterpene content. We hypothesized that the polysaccharide was the basis of bioactivity. To investigate this possibility, we focused on the two major polysaccharides from each species, characterizing them, assessing their antitumor activity and exploring the potential mechanism, involving immune stimulation and gut microbiota alteration.

First, the present study demonstrated that the major polysaccharides in the two species were fundamentally identical. We tested multiple batches of samples to eliminate the possibility of misevaluation caused by complex analytical methods or of differences in individual plants. Focusing on these polysaccharides, several methods were employed to support the conclusion. Firstly, it is indicated that the same fractional polysaccharides from GL had close features to GS in molecular weight distribution and monosaccharides composition profiles. Similar results can be found in the other studies in comparison of crude polysaccharides with close molecular weight distribution patterns by HPSEC-ELSD and obvious bands corresponding to galactose and glucose by HPTLC^12,20^. In addition, studies reported that polysaccharides with different monosaccharides composition and linkages presented variable acid induced hydrolysates-oligosaccharides profiles. Interestingly, we found that oligosaccharides released by mild hydrolyzation of polysaccharides from both species had similar patterns, indicating potentially close structural characters. In further exploration of comparison in structural characterization, the polysaccharides were turned out to share most of the structural consistency in the aspect of IR and NMR spectra patterns and major linkage type’s distributions. To compare the structural similarity, previous effort has been made on using the response to enzymatic digestion of crude polysaccharides from GL and GS^12^. It suggested that polysaccharide from both species were positive to the same enzymes, such as lichenase and β-glucanase, indicating the existence of (1,4)-β-D-glucosidic linkages which were also confirmed in our study (Fig. 3 and Supp. Table 1). Considering the limited kinds of enzymes corresponding to various linkage types, classical and simple approaches-methylation and IR/NMR analysis were used^20–22^.

Furthermore, our animal study revealed that potent and similar antitumor activity against 4T1 were found in mice that had received polysaccharides from two species by oral administration. Similar results have been reported when sarcoma-180 or the same tumor model was treated with the polysaccharide extracts from GL by intraperitoneal or oral administration^22,23^. In general, β-D-glucans, bearing linkages (1 → 3, 1 → 4 or/and 1 → 6) residues proved to be antitumor and immune-enhancing in many reports^24–27^, while the antitumor effect of α-d-glucan is seldom reported^28^. While our result indicated that all the GLW/GSW mainly possessing α-D-anomeric configurations and the GLA/GSA mainly having β-D-anomeric configurations significantly inhibit the tumor growth. An important difference compared to the literatures is that our polysaccharides worked via oral administration while in many other studies the polysaccharides were dosed via *i.p*. injection.

In addition, different from *in vivo* studies, our *in vitro* studies suggested that with the dose of 400 μg/mL all polysaccharides had no cytotoxicity against 4T1 cancer cells. Data from other studies also showed that polysaccharide from GL had little or no direct cancer cell-killing effect^23,24^. Instead, obvious cytotoxicity was observed where macrophages were involved, indicating the important role of macrophage in the interaction. Polysaccharides from two species resembled each other in activating murine macrophages, inducing enhancement of phagocytosis and increase in NO release and cytokines IL-6 and TNF-α production. This may in turn boost the tumoricidal activities. For example, TNF-α can trigger the release of a portfolio of cytokines and activate and recruit immune cells^25^. NO exerts cytostatic effects on tumor cells via NO-dependent pathways^26^. Meanwhile, the generation of NO and cytokine secretion as cellular responses in macrophages was reported to be characterized by activating the toll-like receptor 4 (TLR 4) signaling pathway activated by botanical polysaccharides^27^. Hsu, *et al*.^28^ reported extract of GL polysaccharide- EORP could induce cytokine expression via activation of MAPKs: ERK, JNK, and p38. In addition, Yu. *et al*.^29^ proofed that a purified polysaccharide from *G. atrum* (PSG-1) could promote macrophage acitivities by activating the NF-κB pathways. In our study, the phosphorylation level of phosphorylated MAPKs, such as JNK, p38 and ERK as well as NF-κB proteins in RAW264.7 cells can be increased by treatment with polysaccharides from either GL or GS, suggesting their similarity in possible TLR4 signaling pathway activation.

Our results indicated that polysaccharides from the two species modulated gut microbiota in similar ways. Previous research has suggested that most extraneous polysaccharides are indigestible until they reach the intestine^30,31^. In the gut, they can be fermented by microbes and in turn stimulate the growth and/or activity of some communities, thereby influencing the host’s health^32^. *Firmicutes* and *Bacteroides* are two main advantageous phyla in the gut of mammals. Feeding polysaccharide from GL can alter the shifted ratio of Firmicutes and Bacteroides^33–35^. The alteration of ratio has been reported to be associated with cancer, obesity, diabetes, and other diseases. In agreement with these studies, we found the changed ratio of *Firmicutes* and *Bacteroides* by 4T1 carcinogenesis effect tended to be altered finally with the tumor suppression. In addition to the shift at the phylum level, the polysaccharides changed some important communities in antitumor-related genera. For instance, *Alistipes*, a major short-chain fatty acid producer, recovered and was further enriched after feeding with these fractional polysaccharides. A previous study had revealed that *Alistipe. shahii* played an important role in suppression of tumor growth^36^. In addition, rising abundance of *Prevotellaceae_UCG-001* was observed. Unfortunately, there is little evidence on functions of this strain; however, *Prevotella*, of the family *Prevotellaceae* has been reported to regulate the immune cell population, suppressing tumor growth in mice^37^. Another genus *Bacteroide* seldom found in the control group but which was increased has been reported to take part in the antitumor effects of the CTLA-4 blockade^38^. Although we found some interesting trends, whether the altering and enriching trends in gut community played a role in shrinking the tumors in mice needs further evidence for validation.

In conclusion, all our findings provide scientific evidence that polysaccharides are the common therapeutic basis of Lingzhi.

## Materials and Methods

### Materials and reagents

Fruiting bodies of GL (GL1~8) were purchased from drug stores in Hong Kong, China; fruiting bodies of GS (GS1~7) were purchased from 7 cultivation farms in Guangdong, Shandong and Yunnan province, China. The identification was authenticated by Professor Zhu-Liang Yang at Kunming Institute of Botany, Chinese Academy of Sciences.

LPS (from *Escherichia coli* 0111: B4), Griess reagent (modified), 3-(4, 5-dimethylthiazol-2-yl)-2, 5-diphenyltetrazolium bromide (MTT), Cis-Di-chiorodiamineplatinum (II) (CDDP) and other chemical standards and regents were all purchased from Sigma-Aldrich. Mouse IL-6 E and TNF-α ELISA kit were purchased from eBioscience (San Diego, CA, USA).

### Preparation of polysaccharides

Dried fruiting bodies of GL-2 and GS-2 2 kg for animal test respectively and other batches 300 g were firstly powdered and then defatted by 95% ethanol. And then the defatted samples were exposed to two times of successive extraction with twenty times the volume of hot water for 2 h. The water extract was concentrated and then went to precipitation by ethanol with final concentration 80%. Then we got crude polysaccharides from GL and GS, namely GLP and GSP. The GLP were further separated through the DEAE Fast Flow material-packed column eluted with distilled H_2_O, producing water fraction polysaccharides GLW and successively eluted with 0.5 mol/L NaCl, producing sodium fraction polysaccharides GLA after cutting off molecules lower than 3.5 kDa. The GSW and GSA were obtained with the same procedures.

### **Characterization of polysaccharides**

#### General chemical characterization

The molecular weights (Mw) and distributions of two fractional polysaccharides were estimated by HPGPC analysis, using a previous method^39^. T-series dextran (Mw 1.27, 5.22, 11.6, 23.8, 48.6, 80.9, 147.6, 273, 409.8, and 667.8 kDa) were used as standard molecular markers.

Compositional monosaccharide profiles of GLW, GSW, GLA and GSA were performed by PMP derivatization with appropriate modification^40^. Briefly, 200 μL of individual standard monosaccharide, mixed standard solution or the totally TFA hydrolyzed sample was mixed with 200 μL NH_3_.H_2_O and 100 μL 0.5 mol/L PMP methanolic solution. The mixture was transferred to 70 °C water bath for 30 min. The derivatization was analyzed using gradient elution with 50 mmol/L ammonium formate in aqueous solution with 10% acetonitrile (A) and acetonitrile (B) at a flow rate of 0.45 mL/min: 0~7 min, 5~8% B; 7~14 min, 8~10% B; 14~14.1 min, 5% B on a Waters CORTECS UPLC C_18_ (2.1 × 100 mm, 1.6 μm) with a C_18_ pre-column at 50 °C.

Partial hydrolysates mapping was achieved by HPTLC method. GLW and GSW (10 mg) were treated with 1 mol/L TFA (1 mL) incubated at 90 °C for 2 h; GLA and GSA were similarly treated but incubated for 4 h. The supernatant of hydrolysates was applied on 0.2 mm silica gel 60 HPTLC plates. Plates were developed twice with n-butanol-ethanol-water 5: 3: 2: (v/v)^41^ as mobile phase and colorized with 5% H_2_SO_4_ in ethanol solution, photographed, and scanned.

### Characterization of related structures

All samples as KBr pellets were recorded with a Fourier transform infrared spectrometer to get IR spectra. Meanwhile, ^1^H and ^13^C NMR spectra were recorded on a Bruker Avance 400 spectrometer at 25 °C, at 400 and 100 MHz, respectively, using 40 mg sample in 0.5 mL D_2_O. Water suppression experiment was performed. Chemical shifts are expressed in ppm by reference shift: 0 ppm for ^13^C-NMR (TMS) and 4.80 ppm for ^1^H-NMR (HDO). Data was processed using MestReNova.

Two typical samples of GLW (GLW-2 and GLW-6) and GSW (GSW-2 and GSW-3) were methylated two times; two typical samples of GLA (GLA-2 and GLA-6) and GSA (GSA-2 and GSA-3) were methylated four times, using the method of Needs and Selvendran^42^. Generally, dried sample (10 mg) was fully dissolved in 4 Å molecular sieve-dried DMSO (2 mL) and incubated with NaOH powder (200 mg) for 1 h. Subsequently, 1.5 mL methyl iodide was added. The methylate was hydrolyzed and converted into partially acetylated and partially methylated alditol acetates, and finally analyzed by GC-MS^43^.

### Animal experiments

BALB/C mice (6–8 weeks of age; female: 20.0 ± 2.0 g) were obtained from Tin Hang Technology Limited (Hong Kong, China). The mice were randomly divided into seven groups: normal, negative control (water), positive control (intraperitoneal injection: 4.0 mg/kg CDDP every four days) and polysaccharide treated (gavage daily with 200 mg/kg of GLW-2, GSW-2, GLA-2, GSA-2). The mice in negative group and polysaccharides treated group were treated in advance for six days. And then each mouse in all groups received an injection of 2 × 10^4^ 4T1 cells into the mammary fat pads. Treatment was performed for another consecutive 18 days Body weight was measured in the whole progress. At the end of experiment, feces samples excluding positive control (5 samples from each group) were collected and stored at −80 °C; tumors were excised and weighed. All experimental protocols were approved by the Hong Kong Baptist University Committee on the Use of Human & Animal Subjects in Teaching and Research and conducted in accordance with the guidelines for the use of experimental animals of Hong Kong Baptist University.

### Cell culture

The murine macrophage cell line RAW264.7 and mouse mammary carcinoma cell line 4T1 were obtained from American Type Culture Collection (Manassas, VA, USA) and were propagated in DMEM high glucose medium (Invitrogen Life Technologies, Carlsbad, CA, USA) supplemented with 10% heat-inactivated fatal bovine serum. Cells were cultured at 37 °C in a 5% CO_2_ incubator.

### Cell viability assay

The viability of 4T1 cells affected by the polysaccharides as well as the RAW264.7 mediated cytotoxicity were measured using MTT assay as previous study^44^. Briefly, 4T1 cells (5 × 10^3^ cells/well) were seeded on a 96-well microplate overnight before treatment with GLW-2, GSW-2, GLA-2, and GSA-2 (400 μg/mL) and LPS (500 ng/mL) for 24 h. After incubation, the MTT dye was added. The mixture was incubated for another 4 h in dark. The formazan crystals present in cells were dissolved by dimethyl sulfoxide. The absorbance was read at 570 nm. In assays of cytotoxicity by RAW264.7 cell supernatants induced by the tested polysaccharides on 4T1 cells, RAW264.7 cells (1 × 10^5^ cells/well) were seeded and then exposed to GLW-2, GSW-2, GLA-2, and GSA-2 (400 μg/mL) and LPS (500 ng/mL) for 24 h. 10 μg/ml PolyB was added to the tested polysaccharide samples to exclude the influence of the potential endotoxin contamination^45^. After centrifugation at 1000 rpm for 10 min, the supernatants were added to 96-well plate containing adherent 4T1 cells (5 × 10^3^ cells/well).

### Macrophage-mediated 4T1 cell migration

The scratch assay was performed to explore the activated RAW264.7 cell supernatant’s ability to inhibit migration on 4T1 cells. Briefly, RAW264.7 cells (5 × 10^5^ cells/well) were seeded in a 6-well plate and cultured with DMEM medium containing 2% FBS following a previously described protocol^46^. The monolayers of seeded 4T1 cells (5 × 10^5^ cells/well) in 6-well plate were scratched with a 200 μL pipette tip after growth to 90% confluence. The scratch distance was measured and recorded after incubation for 0, 6, 12 and 24 h.

### Phagocytic activity assay

Seeded RAW264.7 cells (1 × 10^5^ cells/well) on 96-well plates were exposed to 400 μg/mL GLW-2, GSW-2, GLA-2 and GSA-2 with 10 μg/ml PolyB or LPS (500 ng/mL) for 24 h. 0.1% neutral red (100 μL) was added to each well after discarding the supernatant. Then the detailed assay procedure was performed according to a previously reported study^47^.

#### Determination of NO, IL-6 and TNF-α production

RAW264.7 cells (5 × 10^4^ cells/well) were seeded in 96-well plates, incubated overnight. GLW-2, GSW-2, GLA-2 and GSA-2 (400 μg/mL) or LPS (500 ng/mL) were added and incubated for 24 h; 10 μg/ml PolyB was added to the tested polysaccharide samples and LPS. After treatment, the detailed procedure was as previously described^44^.

#### Western blotting assays

RAW264.7 cells (8 × 10^5^ cells/well in 6-well plate) were treated with 400 μg/mL GLW-2, GSW-2, GLA-2 and GSA-2 with 10 μg/ml PolyB or LPS (500 ng/mL) for 30 min. Then, total protein was extracted. 20 μg protein was loaded and separated according to previously reported procedures^44^. Antibodies were used as the manufacturer’s instructions. After incubation with peroxidase-conjugated goat anti-rabbit or anti-mouse secondary antibodies, the immunoreactivity was visualized using an ECL Kit (Amersham Pharmacia Biotech).

#### 16 S rRNA microbial community analysis

The feces samples’ DNA were extracted using the E.Z.N.A. ® Soil DNA Kit (Omega Bio-Tek, Norcross, GA, USA). The V4-V5 regions of the 16 S rRNA gene were PCR-amplified using primers 515 F 5’-barcode-GTGCCAGCMGCCGCGG)-3′and 907 R 5′-CCGTCAATTCMTTTRAGTTT-3′, where barcode is an eight-base sequence unique to each sample. The amplification process was as follows: DNA denaturation step at 95 °C for 2 min, 25 cycles at 95 °C for 30 seconds, 55 °C for 30 s, 72 °C for 30 s, and 72 °C for 5 min. Amplicons were extracted from 2% agarose gels and purified using the AxyPrep DNA Gel Extraction Kit (Axygen Biosciences, Union City, CA, U.S.) and quantified using QuantiFluor™ -ST (Promega, U.S.). Purified amplicons were pooled in equimolar and paired-end sequenced (2 × 250) on an Illumina MiSeq platform according to the instruction. The raw reads were deposited into the NCBI Sequence Read Archive (SRA) database. Raw fastq files were demultiplexed, quality-filtered using QIIME (version 1.17). Operational Units (OTUs) were clustered with 97% similarity cutoff using UPARSE (version 7.1)^48^ and chimeric sequences were identified and removed using UCHIME. The phylogenetic affiliation of each 16 S rRNA gene sequence was analyzed by RDP Classifier against the silva (SSU115) 16 S rRNA database using confidence threshold of 70%^49^.

#### Statistical analysis

Statistical differences between each experimental group were examined by ANOVA, and statistical significance was determined at *p < 0.05, **p < 0.01, ***p < 0.001, ****p < 0.0001. Each experiment was conducted three times or as indicated; all data are expressed as mean ± SD.

### Data availability

All data generated or analysed during this study are included in this published article and its Supplementary Information files.

## Electronic supplementary material

Supplementary information
