# Supplementary material for: Comprehensive comparison of polysaccharides from Ganoderma lucidum and G. sinense: chemical, antitumor, immunomodulating and gut-microbiota modulatory properties
Source: Sci Rep. 2018 Apr 18;8:6172. doi: 10.1038/s41598-018-22885-7 (PMC5906605; doi:10.1038/s41598-018-22885-7)
Supplement: Supplementary file 1 — Supplementary information [file 41598_2018_22885_MOESM1_ESM.pdf]

Comprehensive comparison of polysaccharides from *Ganoderma lucidum* and *G. sinense*: chemical, antitumor, immunomodulating and gut-microbiota modulatory properties

Li-Feng Li[#1], Hong-Bing Liu[#1], Quan-Wei Zhang[1], Zhi-Peng Li[1], Tin-Long Wong[1], Hau-Yee Fung[1], Ji-Xia Zhang[2], Su-Ping Bai[2], Ai-Ping Lu[1], Quan-Bin Han[*1]

[1]*School of Chinese Medicine, Hong Kong Baptist University, Hong Kong, China*

[2]*School of Pharmacy, Xinxiang Medical University, Xinxiang, China*

[*]Corresponding author

[#]These authors contributed equally to this work.

Supplementary Table 1. Main linkage analysis results of typical GLW and GLA from *Ganoderma lucidum* and GSW and GSA from *G. sinense* by methylation and GC-MS

NO	Methylated sugars	Type of linkage	Peak area percentage (≥10%)							
			GLW-2	GLW-6	GSW-2	GSW-3	GLA-2	GLA-6	GSA-2	GSA-3
1	2,3,4,6-M_4-Glc*p*	1- Glc*p*	17.5	15.7	10.2	5.4	21.3	19.4	20.6	22.7
2	2,4,6-M_3- Glc*p*	1,3- Glc*p*	9.4	12.3	8.6	5.2	19.7	22.3	22.8	18.9
3	2,3,6-M_3- Glc*p*	1,4- Glc*p*	16.7	27.3	7.9	6.2	25.8	20.1	15.9	20.3
4	2,3,4-M_3- Gal*p*	1,6- Gal*p*	35.0	21.7	43.6	50.4	-	-	-	-
5	2,3,4-M_3- Glc*p*	1,6- Glc*p*	-	-	-	-	12.9	11.4	19.6	15.6
5	2,4-M_2- Glc*p*	1,3,6- Glc*p*	< 10	<10	<10	<10	14.6	15.8	13.1	10.8
6	3,4-M_2-Gal*p*	1,2,6-Gal*p*	7.1	4.4	11.5	12.8	-	-	-	-

Comprehensive comparison of polysaccharides from *Ganoderma lucidum* and *G. sinense*: chemical, antitumor, immunomodulating and gut-microbiota modulatory properties

Li-Feng Li[#1], Hong-Bing Liu[#1], Quan-Wei Zhang[1], Zhi-Peng Li[1], Tin-Long Wong[1], Hau-Yee Fung[1], Ji-Xia Zhang[2], Su-Ping Bai[2], Ai-Ping Lu[1], Quan-Bin Han[*1]

[1]*School of Chinese Medicine, Hong Kong Baptist University, Hong Kong, China*

[2]*School of Pharmacy, Xinxiang Medical University, Xinxiang, China*

[*]Corresponding author

[#]These authors contributed equally to this work.

Supplementary Figure legends

Supplementary Figure 1. HPGPC chromatograms of molecular distribution of GLWs (a), GSWs (b), GLAs (c) and GSAs (d).

Supplementary Figure 2. Monosaccharide composition profiles of GLWs (a), GSWs (b), GLAs (c) and GSAs (d) by UPLC-UV (λ=245 nm) after 1-phenyl-3-methyl-5-pyrazolone (PMP) derivatization. Mixed standards (Mix Std) contain the following saccharides: Mannose (Man); Ribose (Rib); Rhamnose (Rha); Glucuronic acid (GlcA); Galacturonic acid (GalA); Glucose (Glc); Galactose (Gal); Arabinose (Ara); Fucose (Fuc).

Supplementary Figure 3. HPTLC fingerprint profiles of TFA-induced partially hydrolyzed GLWs (a) and GSWs (b), GLAs (c) and GSAs (d). Samples were applied on 0.2 mm silica gel 60 HPTLC plates (Merck, Germany) with an automatic TLC sampler (CAMAG, Switzerland). Then the plate was developed with n-butanol–ethanol–water 5: 3: 2: (v/v) and colorized with 5% H_2SO_4 in ethanol solution and heated to make bands colored clearly. Then the plate was photographed by a TLC visualizer under white light.

Supplementary Figure 4. IR spectra of GLWs (a), GSWs (b), GLAs (c) and GSAs (d).

Supplementary Figure 5. Full-length blots of tested proteins in in RAW264.7 macrophages cells. The related protein is marked, and other blots presented in the whole film is from other experiments. (a) phosphorylation of ERK and ERK; (b) phosphorylation of JNK and JNK; (c) p38 MAPK, p65 and β-actin; (d) phosphorylation of p38 MAPK; (e) phosphorylation of p65.

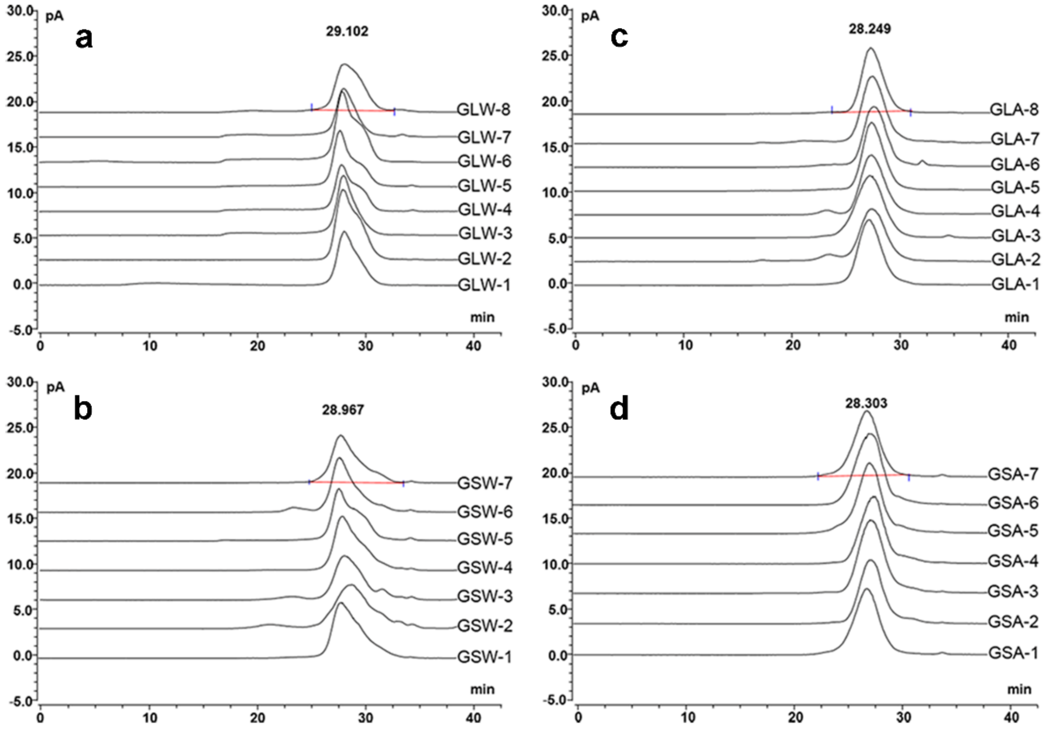

Supplementary Figure 1

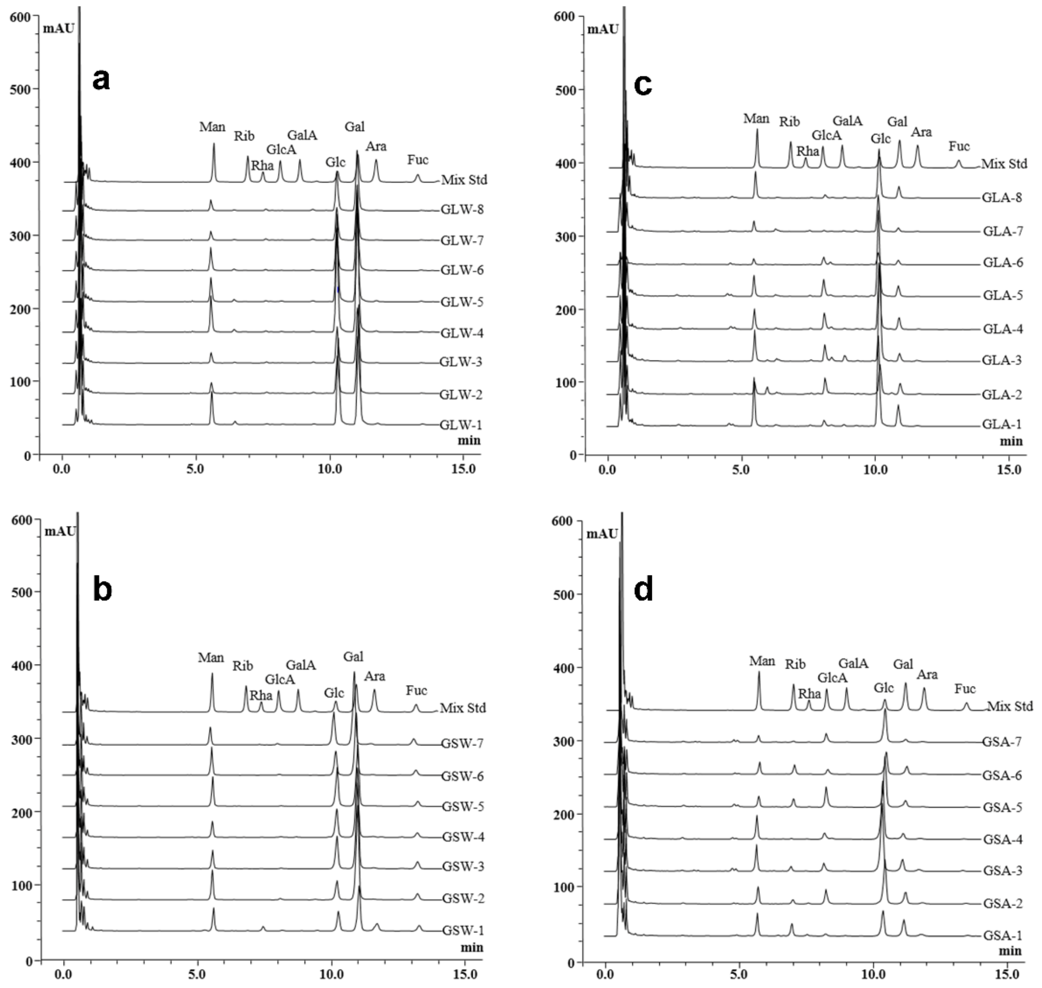

Supplementary Figure 2

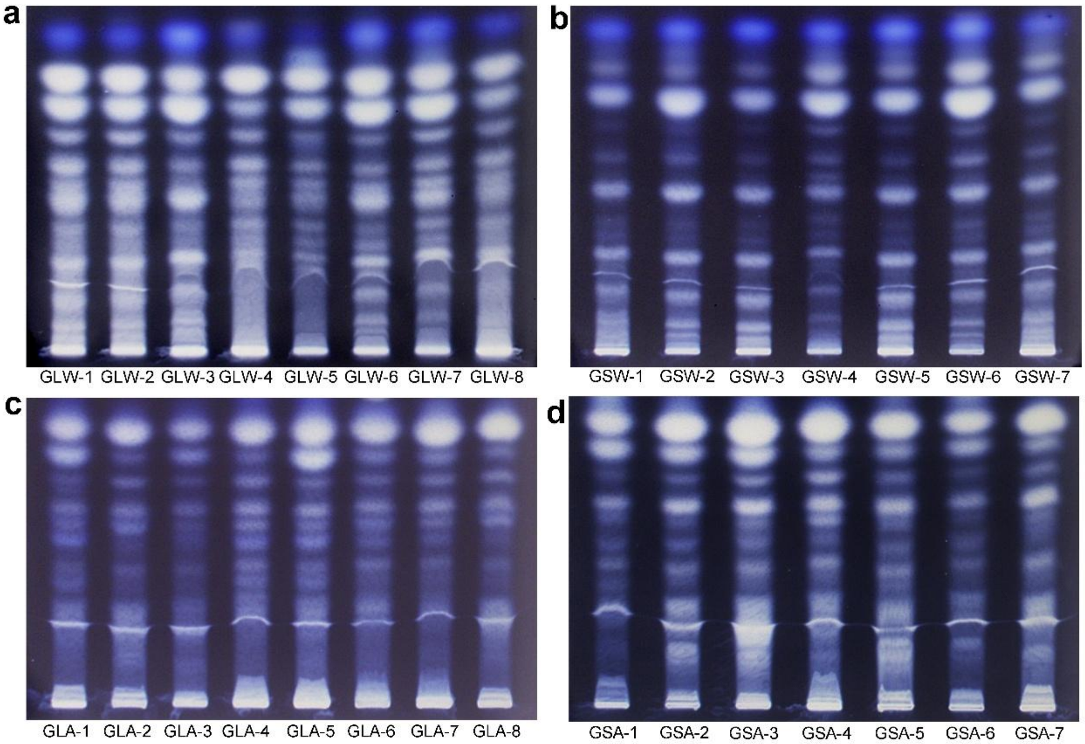

Supplementary Figure 3

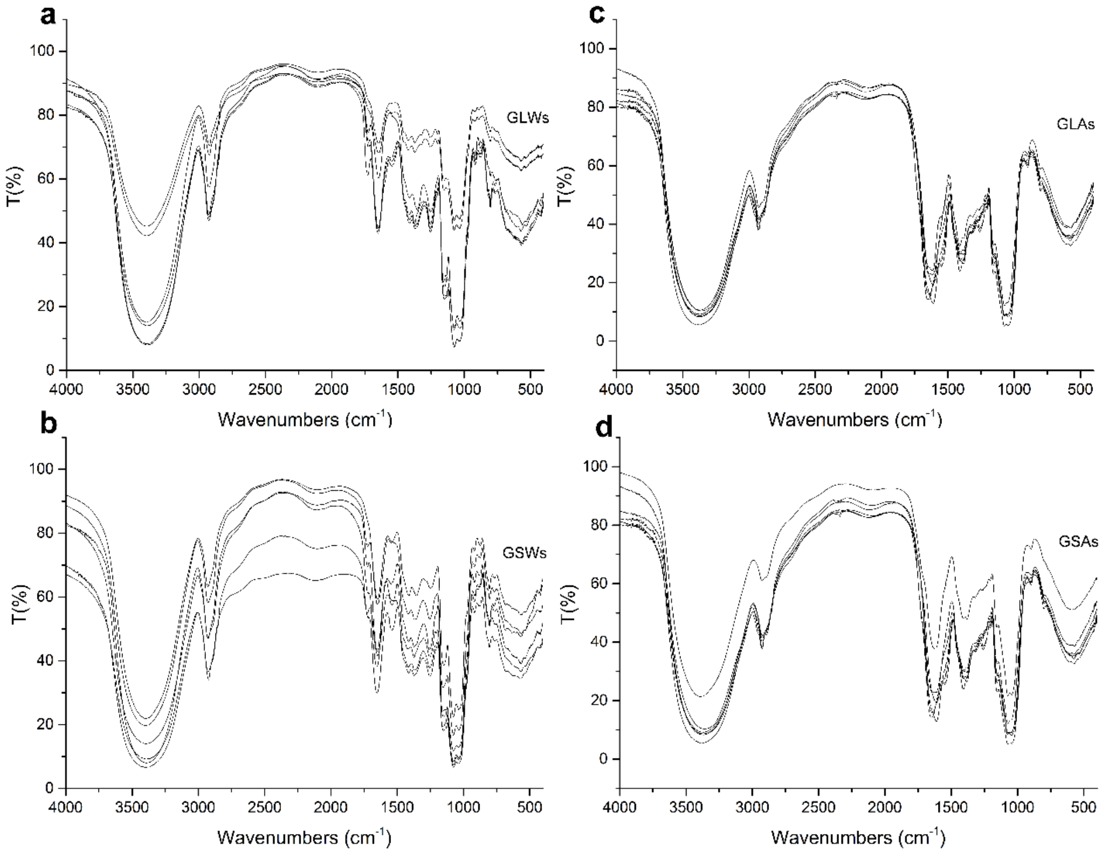

Supplementary Figure 4

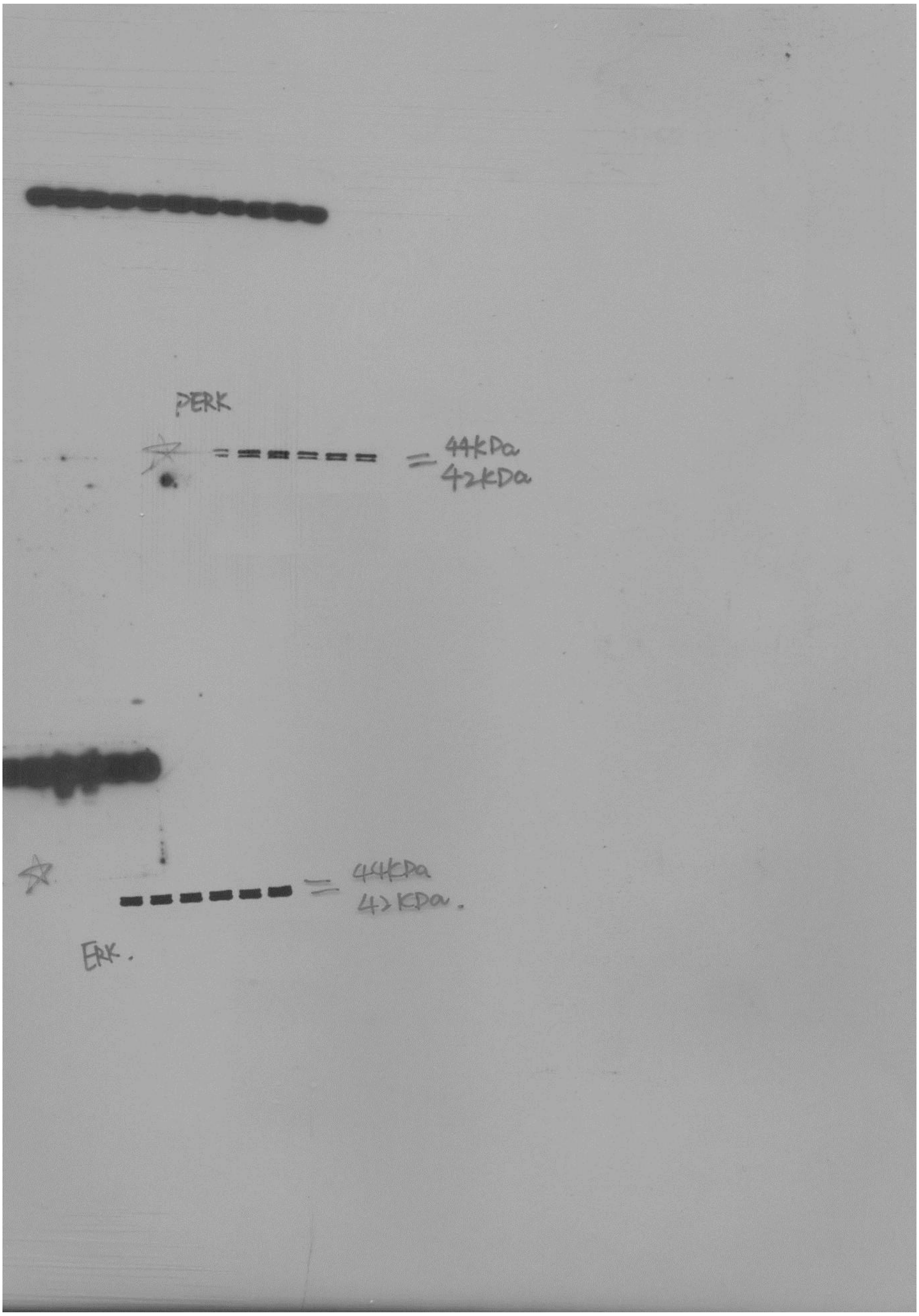

Supplementary Figure 5 a

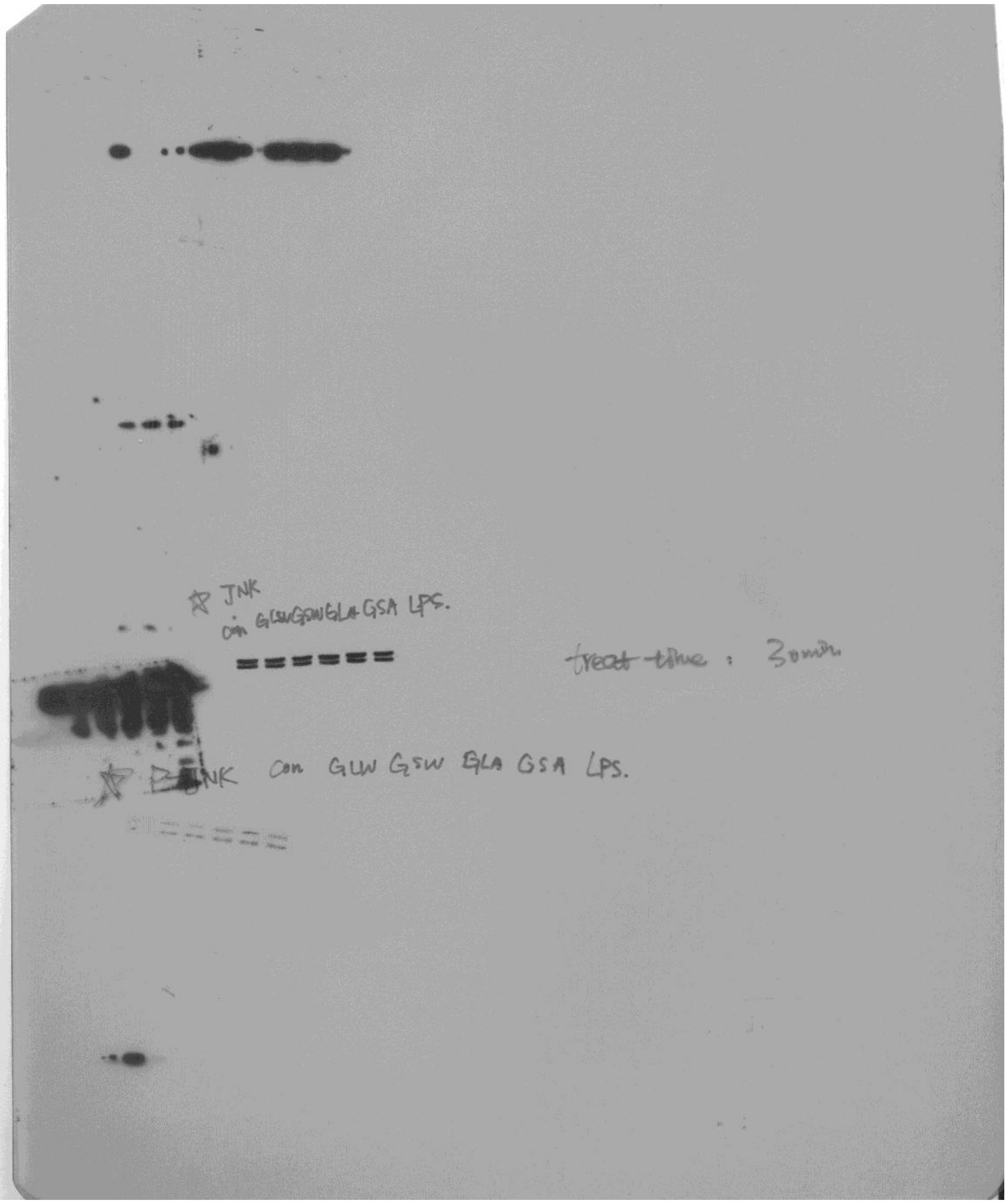

Supplementary Figure 5 b

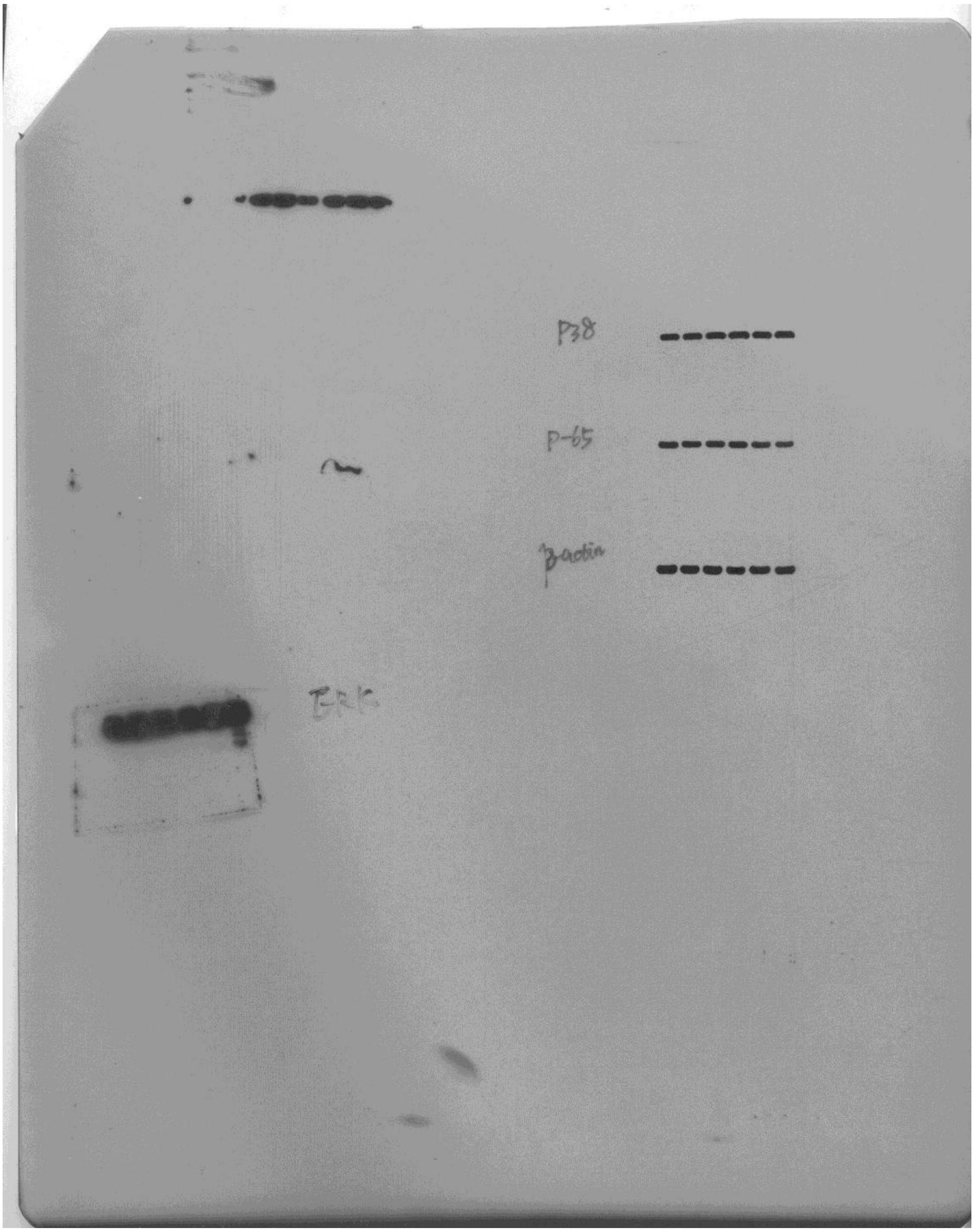

Supplementary Figure 5 c

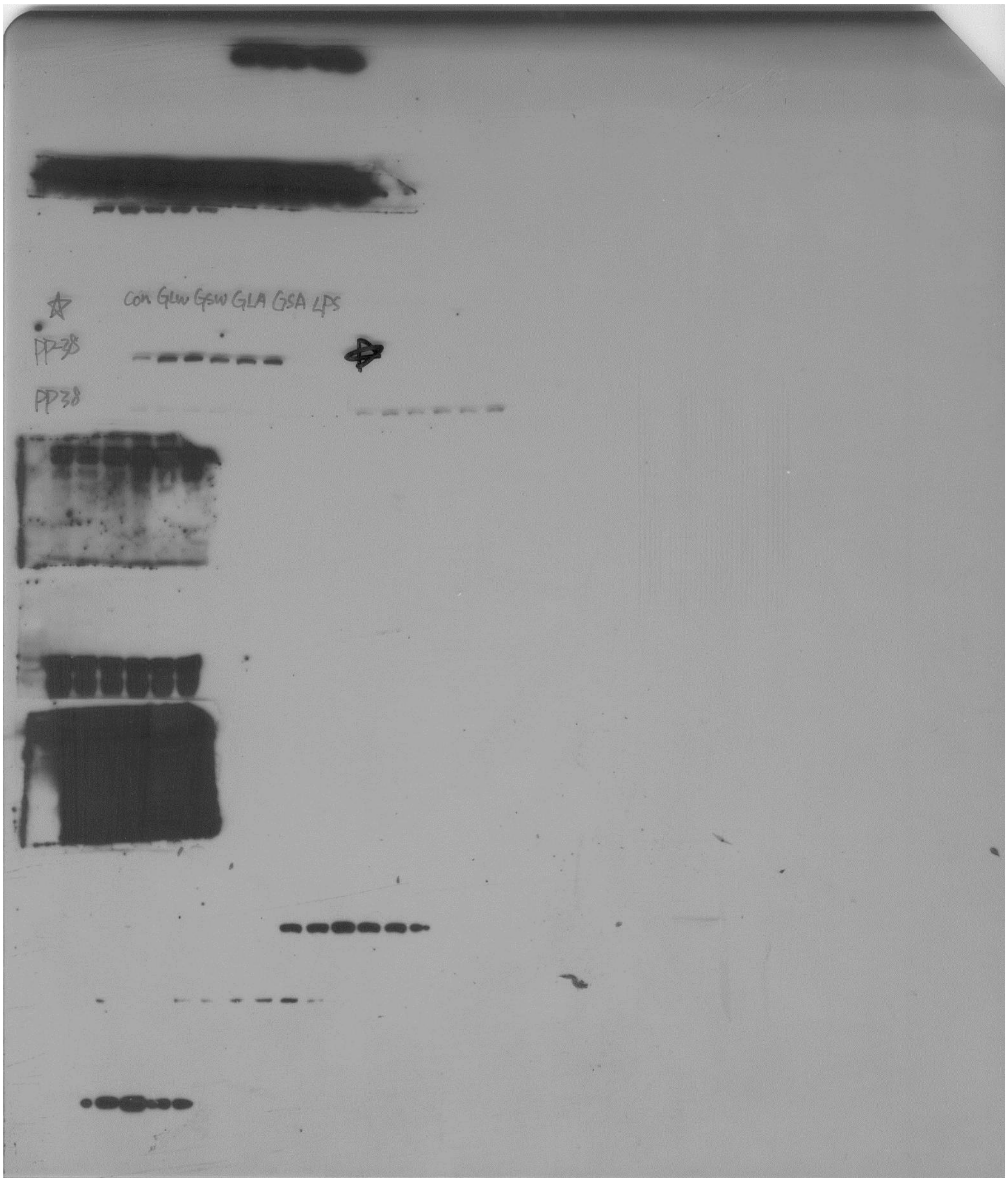

Supplementary Figure 5 d

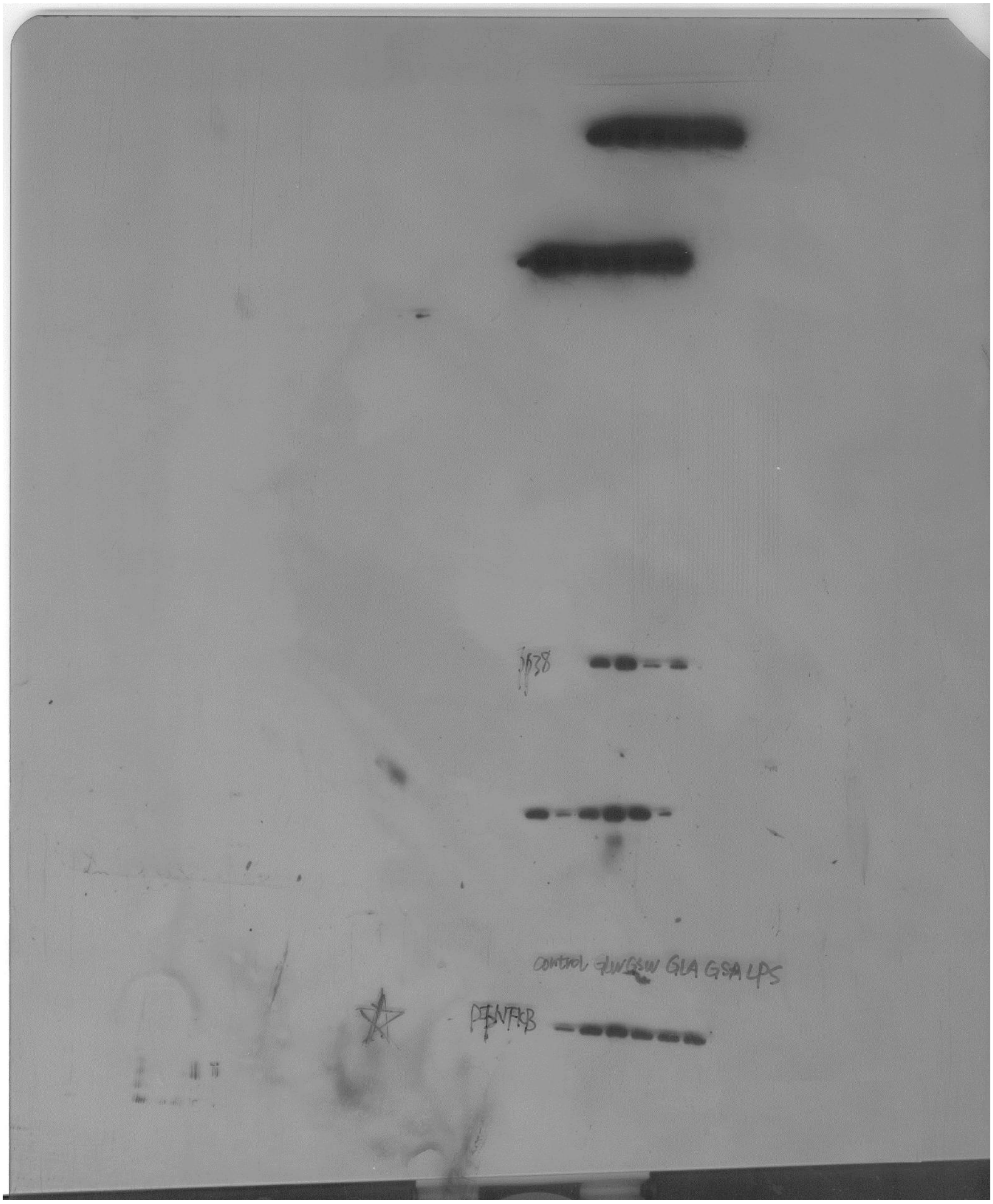

Supplementary Figure 5 e
